# Integrating differential privacy in deep reinforcement learning for sepsis treatment with pulmonary implications

**DOI:** 10.3389/fmed.2025.1590824

**Published:** 2025-04-17

**Authors:** Shuling Wang, Feng Yang, Suixue Wang, Rongdao Sun

**Affiliations:** ^1^Department of Neurology, Haikou Affiliated Hospital of Central South University Xiangya School of Medicine, Haikou, China; ^2^School of Computer Science and Technology, Hainan University, Haikou, China

**Keywords:** pulmonary diseases, sepsis treatment, artificial intelligence, deep reinforcement learning, differential privacy, clinical treatment plan

## Abstract

Pulmonary diseases, such as pneumonia and lung abscess, can trigger sepsis, while sepsis-induced immune dysfunction exacerbates Pulmonary tissue damage, creating a vicious cycle. Therefore, designing a safe and effective clinical treatment planning method for sepsis is critically significant. In recent years, deep reinforcement learning (DRL), as one of the artificial intelligence technologies, has achieved remarkable results in the field of sepsis treatment. However, DRL models may be attacked due to their sensitive training data and their high commercial value, especially with the increasing number of DRL models being released on the Internet. Consequently, protecting the “privacy” of DRL models and training data has become an urgent problem. To address this issue, we propose a differential privacy-based DRL model for sepsis treatment. Furthermore, we investigate the impact of differential privacy mechanisms on the performance of the DRL model. Experimental results demonstrate that integrating differential privacy into DRL models enables clinicians to design sepsis treatment plans while protecting patient privacy, thereby mitigating lung tissue damage and dysfunction caused by sepsis.

## 1 Introduction

Sepsis is a life-threatening condition caused by a dysregulated host response to infection, resulting in systemic inflammation and organ dysfunction (1). The primary sources of infection include the Pulmonary (40–60%), abdomen, urinary tract, and bloodstream (2). Inflammatory factors released during sepsis, such as tumor necrosis factor-α and interleukin-1β, disrupt the alveolar-capillary barrier, leading to pulmonary edema, respiratory failure, and progression to acute respiratory distress syndrome (ARDS) (1, 2). Additionally, excessive inflammatory mediator release induces brain microvascular endothelial injury and coagulation dysfunction, increasing the risk of cerebral microcirculation disorders and cerebral infarction. Therefore, designing a safe and effective clinical treatment plan for sepsis is of critical importance.

Recently, with the development of computer computing power, deep reinforcement learning has achieved surprising achievements in real-world scenarios, including medical care, chess, and games. We have witnessed recent breakthroughs in reinforcement learning, such as the emergence of the Deep Q-Network (3), which surpassed human levels in three of the seven games on the test. In 2016, Komorowski et al. (4) first proposed using reinforcement learning (RL) to address challenges in the medical field, They propose a discrete MDP to provide optimal treatment options for terminally ill patients in the intensive care unit, demonstrating the great potential of reinforcement learning in providing the individualized treatment.

**Figure 1:**
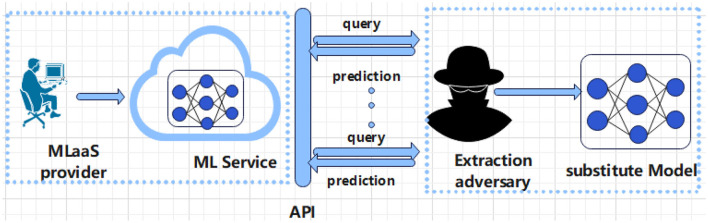
Schematic diagram of model extraction attack.

Reinforcement learning, like other machine learning methods (5–7), requires training in representative data, which often contains sensitive personal information, to obtain ideal models. Ideally, sensitive personal privacy should not be leaked during the process of training a machine learning model. In other words, the parameters of the machine learning model should learn general patterns (People with a sweet tooth are more likely to develop diabetes), not some specific training samples (he has diabetes). But deep reinforcement models can remember a user's private information “inadvertently”. Zhang et al. (8) argued that deep models, such as convolutional neural networks, can accurately memorize arbitrary labels of training data. Shokri et al. (9) proposed a member inference attack: Given a sample, it can be inferred whether the sample is in the training data set of this model. Even if the parameters and structure of the model are poorly known, this attack is still effective. Unfortunately, the same risk exists for reinforcement learning, and Pan et al. (10) showed that RL agents can leak information about the environment, which indicated the risk of leaking the privacy of users in the training data. Both training data, the structure and parameters of the model should also be protected. The commercial value of the model makes it likely to be subjected to Model Extraction attacks (The schematic diagram of a model extraction attack is shown in Figure 1). The recent trend is machine learning as a service, which means that a proven model can serve a large number of users and generate significant revenue for the company. For example, the autonomous driving company TuSimple relies on its driverless transportation solutions which are expected to bring in over a billion dollars in annual earnings. Tramer et al. (11) proposed the model extraction attacks, which can obtain a model with close to perfect fidelity under the condition that he only has access to the black box of the model without model parameters or training data.

To prevent privacy leak issues with deep reinforcement learning for sepsis treatment, this paper proposes a Privacy-Preserving deep Q network based on differential privacy for Sepsis Treatment. In detail, the purpose of this study and our main contributions are:

1. We propose a privacy-preserving deep reinforcement learning model. In particular, we add perturbations to gradient descent using Gaussian noise and calculate the privacy budgets.
2. We analyzed the performance of the model in treating patients with different severities of sepsis, counted the choice of actions of the model, and summarized the effect of the inclusion of differential privacy on the model strategy, and we explained the reasons for this occurrence.
3. We evaluate the performance of a deep reinforcement learning model for the treatment of sepsis at different privacy budgets, and analyze its impact on the deep reinforcement learning model strategy and patient mortality. Our experiments show that differential privacy can be used to protect deep reinforcement learning models with good performance at reasonable privacy budgets.

## 2 Related work

Privacy protection for machine learning can be divided into three categories according to the protection technology: homomorphic encryption, multi-party secure computing, and differential privacy.

Homomorphic encryption and multi-party secure computing are cryptographic methods that protect data privacy during computing. Sun et al. (12) used approximate homomorphic encryption to solve the problem of untrustworthy outsourcing servers, prevented private health data from being leaked or altered, and provided secure treatment decisions for patients. Liu et al. (13) proposed a privacy-preserving reinforcement learning framework that uses encryption to protect the privacy of patients' current health status and treatment decisions and provides patients with a secure dynamic treatment plan. Xue et al. (14) prevented the privacy of historical electronic medical records (EMRs) from being leaked during training by additively homomorphic encryption schemes to provide personalized treatment plans.

Homomorphic encryption and multi-party secure computing can effectively defend against Reconstruction Attacks during model training but cannot defend against member inference Attacks. Therefore, it is necessary to introduce differential privacy to protect reinforcement learning, and relevant research has been carried out. Wang et al. (15) introduced differentially private Q-learning in the continuous state space to make adjacent reward functions indistinguishable, which can protect the reward information from being exploited by methods such as inverse reinforcement learning. Gajane et al. (16) suggested two methods to solve the Corrupt Bandits problem and discussed the application of the algorithms in privacy protection. Using local differential privacy, the algorithm is suitable for protecting user privacy in recommender systems. Basu et al. (17) introduced and unified privacy definitions for the multi-armed bandit algorithms; they used a unified graphical model to represent the framework, used it to connect the privacy definitions, and derived the regret lower bounds on the regret of bandit algorithms satisfying these definitions. Ono et al. (18) implemented distributed reinforcement learning under local differential privacy(LDP). Agents do not submit deliverable data but submit perturbed gradients. They propose the Laplacian method and random projection method to introduce randomness to satisfy the LDP distribution gradient to prevent information leakage. We focus on reinforcement learning for centralized learning, using Gaussian mechanisms to add randomness to gradients and analyze its application in sepsis treatment.

## 3 Preliminaries

In this section, we briefly introduce the definition of differential privacy and outline the fundamentals of reinforcement learning.

### 3.1 Differential privacy

Differential privacy is a strictly proven privacy-preserving technology. This definition was first proposed by Microsoft's (19). It is defined according to the application-specific concept of adjacent databases, that is, two datasets differ by only one record.

Definition 1: (ε, δ)−Differential privacy (20). A random algorithm *M*:*D* → *R* satisfies (ε, δ)−differential privacy if and only if for any adjacent data set d, d′ ∈ D and any output S ⊆ R differing by only one piece of data, the following conditions are satisfied:


(1) $$\begin{array}{l} \mathrm{Pr}\left[\text{M}\left(\text{d}\right)\epsilon \text{S}\right]\leq e^{\varepsilon }\mathrm{Pr}\left[M\left(d^{\prime }\right)\epsilon S\right]+\delta \end{array}$$

Where, M(d) and M(d') represent the output of the algorithm M on the data sets d,d' respectively; Pr is the output probability of the algorithm; ε is the privacy budget, which is used to control the level of privacy protection. The smaller the ε, the stronger the privacy protection ability provided; δ is another privacy budget, representing the probability that the tolerable privacy budget exceeds ε.

Definition 2: [Global sensitivity (21)]. Given a function *f*:D → *R*^*d*^, for any two adjacent datasets D and D′, the global sensitivity of f is defined as:


(2) $$\begin{array}{l} \text{G}{\text{S}}_{f}{=}_{D,D^{\prime }}^{\max}|f\left(D\right)-f\left(D^{\prime }\right)| \end{array}$$

Definition 3: [l2-global sensitivity (21)]. The l2-global sensitivity of a function f is the maximum L2 norm of the difference between f(D) and *f*(*D*′), defined below:


(3) $$\begin{array}{l} \text{L}2_{f}{=}_{D,D^{\prime }}^{\max}|f\left(D\right)-f\left(D^{\prime }\right)| \end{array}$$

Definition 4: [Gaussian mechanism (20)]. where $\left(0,S_{f}^{2}\cdot \sigma^{2}\right)$ is the normal (Gaussian) distribution with mean 0 and standard deviation *S*_*f*_σ. defined below:


(4) $$\begin{array}{l} \text{M}\left(\text{D}\right)≜f\left(\text{D}\right)+N\left(0,S_{f}^{2}\cdot \sigma^{2}\right) \end{array}$$

Theorem 1 (20). Let ε ∈ (0, 1), the Gaussian mechanism with parameter $\sigma >2\sqrt{\ln(1.25/\delta \text{L}2_{f}/\varepsilon }$is (ε, δ)−DP. According to Theorem 1, the Gaussian mechanism can achieve a (ε, δ)−DP guarantee as long as those parameters ϵ, δ and σ meet the above inequality conditions.

Differential privacy can be used in machine learning because of its special properties:

Property 1 (post-processing immunity). For the same dataset D, if the mechanism M satisfies ϵ-differential privacy, then for any random algorithm A (not necessarily satisfying the definition of differential privacy), the new mechanism *M*′ = *A*(*M*(*D*)) still satisfies ϵ-differential privacy.

Property 2 (sequence compositionally). If a series of algorithms M1, M2, ..., Mk satisfies (ε, δ)−differential privacy, then for the same data set D, the combined algorithm [M1(D), M2(D),…, Mk(D)] provide (*kϵ, kδ*)-differential privacy protection.

Differential privacy technology makes it impossible for malicious adversaries to infer sensitive information of training data or models even if they can obtain algorithm services and output through the algorithm.

### 3.2 Reinforcement learning

**Figure 2:**
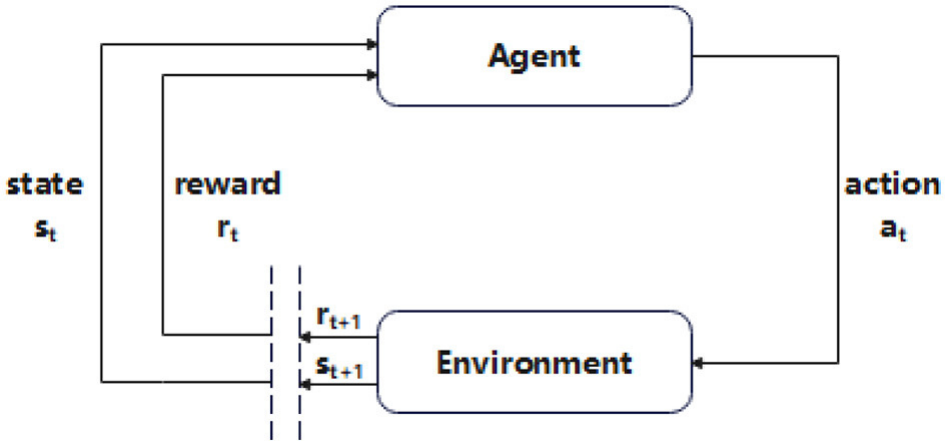
The interaction diagram between the agent and the environment.

Reinforcement learning is a branch of machine learning that allows computers to interact with their environment and is used extensively to solve sequential decision problems. The “interaction” of the agent is realized through a predefined action set A = {*a*_1_, *a*_2_⋯}, which contains all possible actions. The agent first observes the current state, then performs the selected action, and obtains environmental feedback, and the learning process is to repeat the above process. This learning process establishes a loop between the environment and the agent, as shown in Figure 2.

The standard theory of reinforcement learning is defined by the Markov Decision Process (MDP), where the immediate reward depends only on the present state and action.

#### 3.2.1 Markov decision process (MDP)

The Markov decision process can be represented by a tuple < S, A, P, R, γ>, where S is a set of state/observation spaces of the environment; A is the action set of the agent; P is a transition probability function P(*s*_*t*+1_∣*s*_*t*_, *a*_*t*_), which represents the probability of transitioning to s_*t*+1_ ∈ S if the agent takes action a ∈ Ain state *s* ∈ S; R is a reward function, and the immediate reward is *R*_*t*_ = *R*(*s*_*t*_, *a*_*t*_); γ ∈ [0, 1] is a discount factor, which determines the degree of influence of future rewards on the current. The purpose of reinforcement learning is to maximize the expected return $E\left[\underset{t}{\sum }\gamma^{t}r_{t}\right]$ by optimizing the strategy. The optimal value function is defined as:


(5) $$\begin{array}{l} V^{*}\left(s\right)=\underset{\pi }{\max}E\left[\underset{t}{\sum }\gamma^{t}r_{t}|S_{0}=s,\pi \right] \end{array}$$

The action-value function is introduced to estimate the expected revenue of the strategy π. This value is the mathematical expectation of return, approximated by the Bellman expectation equation.

#### 3.2.2 Bellman equation

The Bellman equation, also known as the Bellman expectation equation, is used to calculate the expectation of the value function on the mining trajectory under the guidance of the strategy given the strategy π. The optimal state-action value function is defined as:


(6) $$\begin{array}{l} Q^{*}\left(s,a\right)=\underset{\pi }{\max}E\left[\underset{t}{\sum }\gamma^{t}r_{t}|S_{0}=s,a_{0}=a,\pi \right] \end{array}$$

The above equation satisfies the Bellman equation can be written as:


(7) $$\begin{array}{l} Q^{*}\left(s,a\right)=r\left(s,a\right)+\gamma \underset{a^{\prime }}{\max}E\left[Q^{*}\left(s^{\prime },a^{\prime }\right)\right] \end{array}$$

#### 3.2.3 Deep reinforcement learning

Deep reinforcement learning combines the advantages of deep learning and reinforcement learning. The deep neural network has strong data expression ability, and the action-value function is approximated by the neural network, so it can be written as:


(8) $$\begin{array}{l} Q\left(\text{s},\text{a},\theta \right)\approx Q^{*}\left(s,a\right) \end{array}$$

where θ is the parameter of the neural network.

## 4 Our approach

This section describes the main components of our study, including the deep reinforcement learning model for the treatment of sepsis, the differentially private gradient descent algorithm, and the computation of the privacy budgets of differential privacy.

### 4.1 DP-DQN

This section introduces our proposed DP-DQN algorithm, which outlines how to incorporate privacy protection in reinforcement learning based on the DQN model and calculate the privacy budgets. gradient perturbation can achieve DP guarantee even for nonconvex objectives, so this paper chooses to take the gradient perturbation approach. The pseudocode of Algorithm 1 outlines the basic steps of our algorithm.

**Algorithm 1:**
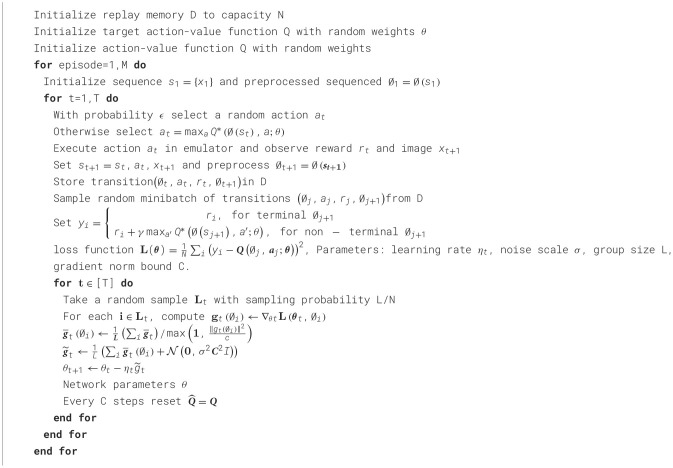
DP-DQN algorithm.

Norm clipping. We need to limit the influence of every single example on the gradient ${\tilde{g}}_{t}$ to provide the differential privacy guarantee of Algorithm 1, for this we need to perform gradient clipping, in which we use the L2 norm to measure, define C as the threshold, the gradient is greater than The gradient of C will be clipped to C, the gradient smaller than C will retain the original value, then ${\tilde{g}}_{t}$ will be replaced by $\max\left(1,\frac{||g_{t}\left(\emptyset_{i}\right)|{|}^{2}}{c}\right)$.

Crafting Noisy Gradient. We choose to add Gaussian noise, which is different from Ono et al. (18) adding Laplacian noise to LDP. The sum of the two Gaussian functions is a Gaussian function, so the impact of privacy mechanisms on statistical analysis is easier to understand and correct. Gaussian noise achieves relaxed (ε, δ)−differential privacy.

Privacy accounting. The main problem after adding noise is how to calculate the overall privacy budgets. Differential privacy is compostable, which means that we can calculate the privacy budgets by accumulating the privacy budgets as the training number progresses. Each step of training requires adding noise to the multi-layer gradients, and Privacy accounting needs to accumulate the budgets corresponding to all these gradients. We use Moments accountant (22) to calculate Privacy accounting, which provides a more rigorous calculation method than the strong composite theorem. It proves that there are constants *c*_1_ and *c*_2_, given sampling probability q=L/N and number of steps T, for any $\varepsilon <{\text{c}}_{2}{\text{q}}^{2}\text{T}$ and δ>0 and $\sigma \geq C_{2}\frac{q\sqrt{T\log\left(1/\delta \right)}}{\epsilon }$, Algorithm 1 satisfies (ε, δ)−differential privacy. Through this theorem, the privacy budgets in Algorithm 1 can be monitored in real time.

### 4.2 Building the deep reinforcement learning model

This section mainly describes building deep reinforcement learning models and environments for the treatment of sepsis. This scheme replicates the work of Raghu et al. (23) with some improvements.

#### 4.2.1 Screening patients with sepsis

The patients' data were obtained from the MIMIC-III (Medical Information Mart for Intensive Care) dataset, a large public database of patients in the intensive care unit at Beth Israel Women's Dickens Medical Center between 2001 and 2019. According to the latest criteria defined by Singer et al. (24), sepsis is defined as a suspected infection (prescribing antibiotics and collecting body fluids for microbial cultures) with evidence of organ dysfunction, as measured by a Sequential Organ Failure Assessment (SOFA) score greater than or equal to 2 to define. We followed Sepsis-3 criteria to screen patients with sepsis.

#### 4.2.2 State space

Patient histories were divided into 4-h windows, timed from 24 h before the diagnosis of sepsis to 48 h after the onset of sepsis. According to the research of Johnson et al. (25), 46 physiological indicators of patients were selected, patients with too many missing physiological indicators were excluded, and a 47 × 1 feature vector of each patient at each time step was obtained. These feature vectors will be used to represent the current body state of the patient, which is the S in the MDP. The dataset is split into 80% training and validation sets and 20% test sets. See the appendix for details.

#### 4.2.3 Action space

We define the same discrete action space as Raghu et al. (23). Our action consists of doses of two drugs: intravenous (IV) fluids and vasopressors (VP). In the cohort, IV and VP usage were recorded for each patient every 4-h window, the effect space was discretized into quartiles for each drug, and the quartiles for each drug at each time step were transformed is an integer representing its quartile, plus an action space with a dose of 0. This resulted in a 5 × 5 action space, with an action of (0,0) indicating no treatment and an action of (4,4) representing the highest quartile of fluid and vasopressor doses.

#### 4.2.4 Rewards

We focus on patient survival, so we choose to set a sparse reward, giving the agent a +15 reward if the patient survives at the end, a -15 reward if he dies, and a 0 reward otherwise.

#### 4.2.5 Model architecture

The original Deep Q-Networks have obvious shortcomings in that the q-values are often overestimated, so we use an improved version of the model. The model uses the Dueling Double-Deep Q Network architecture. Compared to the ordinary Deep Q-Networks, the Double-Deep Q Network decouples the two steps of target Q-value action selection and target Q-value calculation, and no longer finds the maximum Q-value of each Dueling-Deep Q Network divides the model output q-value into value stream and dominance function stream. The value stream represents the quality of the current state, while the dominance stream represents the quality of the selected action. In this experiment, then, the value stream represents the current physiological state of the patient, and the dominance stream represents the dose of the agent. To accelerate convergence, the model uses preferential empirical replay.

## 5 Experimental results and discussion

This section presents the performance of a deep reinforcement learning model based on differential privacy for sepsis treatment. We analyze the impact by setting up comparison experiments in which we evaluate the learning efficiency, patient survival, and the trade-off between privacy and efficiency, and show here that our approach is effective and feasible.

### 5.1 Action

The strategy of the strengthened model is reflected by the actions, and we analyze the changes brought by differential privacy on the action selection aspect of the model. We take the model satisfying (8, 10^−6^)−differential privacy as an example.

**Figure 3:**
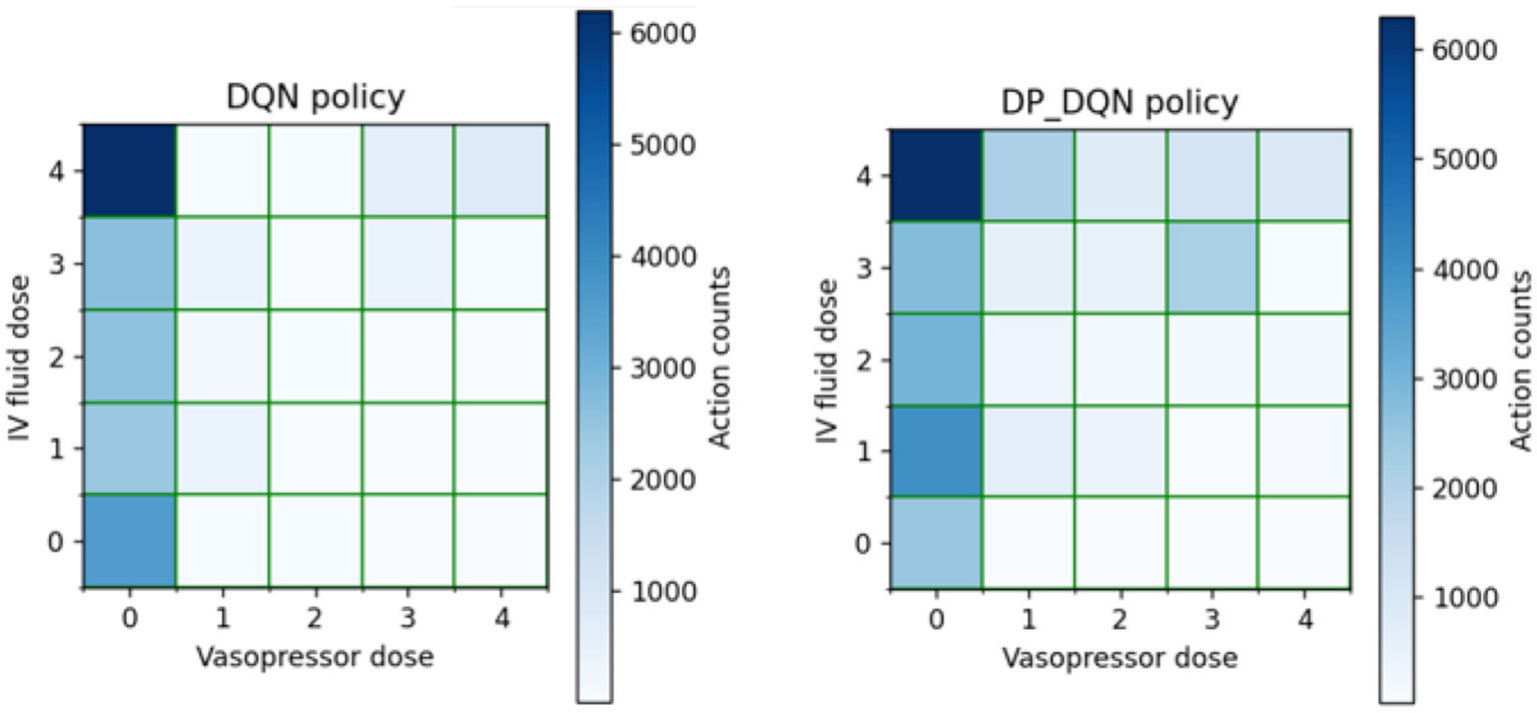
2D histograms were used to represent the discrete action space. (0,0) indicates no treatment and (4, 4) represents the highest quartile dose of fluid and vasopressors.

Figure 3 depicts the overall action selection of the DQN and DP-DQN, which are similar but not identical, suggesting that the agent's strategy produced a change that caused it to select a different sequence of actions at the time of treatment. However, both algorithms follow a rule that less pressurized drugs are prescribed, indicating that DP-DQN can also learn this strategy. Less prescribing of vasopressors can be explained clinically by injecting vasopressors to increase the patient's mean arterial pressure, but many patients with sepsis do not have low blood pressure and thus do not need vasopressors.

We qualitatively analyze the performance of the algorithm in treating patients with different states, and we classified patients as critical according to SOFA, with SOFA scores < 5 for low SOFA, < 15 and > 5 for medium SOFA, and > 15 for high SOFA at the current time step. This is to understand the performance of the model on different severity subcohorts.

**Figure 4:**
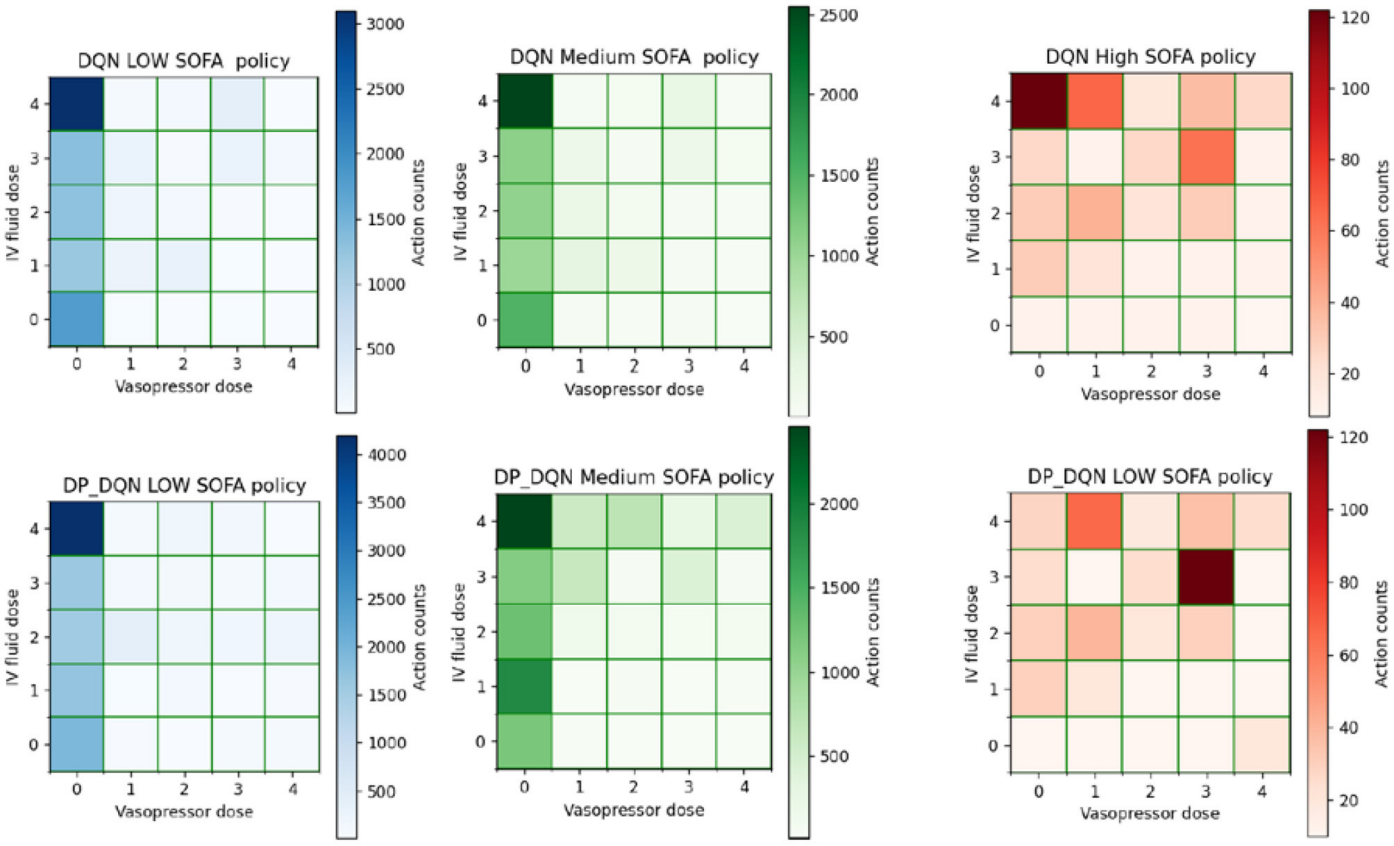
Showing the action selection of the model on different severity subcohorts.

By observing Figure 4, we found that the strategies of DQN and DP-DQN did not change greatly in treating patients with low SOFA and high medium SOFA, with most of the actions concentrated at (0,4) and almost no vasopressors drugs prescribed. In the case of high SOFA patients, the strategies of the DQN and DP-DQN were far apart, with the DQN actions still concentrated at (0,4) and the DP-DQN actions concentrated at (3,3), and the DP-DQN agent started to give larger doses of vasopressors, which was similar to the strategy of the physicians, who mostly concentrated at (4,4) when dealing with this type of patients.

In the work of Neelakantan et al. (26), the authors demonstrated empirically that adding noise encourages active exploration of the parameter space and gives the model more chances to escape local minima or to quickly pass the early learning smooth phase. We argue that this also holds for reinforcement learning, where the perturbation of the gradient makes the model more exploratory and the model more likely to explore different actions. Different strategies will also lead to a different performance in terms of mortality.

Collectively, the deep reinforcement learning model satisfying (8, 10^−6^)−differential privacy is still able to learn good strategies, even to the extent that it learns strategies that are not learned by the original model.

### 5.2 Learning efficiency

We measure the learning efficiency by how many steps the agent can reach the target survival rate in training. To measure it, we introduce a performance metric termed the First Achieve Episode (FAE), which is defined as:


(9) $$\begin{array}{l} \begin{array}{c} \text{FAE}=\min\left\{\text{episode}|\text{episode}\in \left[\text{Episode}\right],\text{Survival rate}\geq \phi \right\} \end{array} \end{array}$$

We compared FAE in different ϵ cases, screening patients with moderate SOFA as the data set. In our experiments, we found that in patients with small SOFA values, mortality did not change significantly with dose, and the mortality rate of patients was maintained at a low level. At the same time, there was no clear pattern in patients with higher SOFA values, and there were fewer patients with high SOFA, which made it difficult for the model to learn a stable strategy. Therefore, the experiment selected the data of patients with moderate SOFA as the dataset, which would make the results more representative. We also compared the effect of whether to include preferential experience replay or not, and we selected the median of 24 experiments considering the possible effect of uneven sampling.

**Figure 5:**
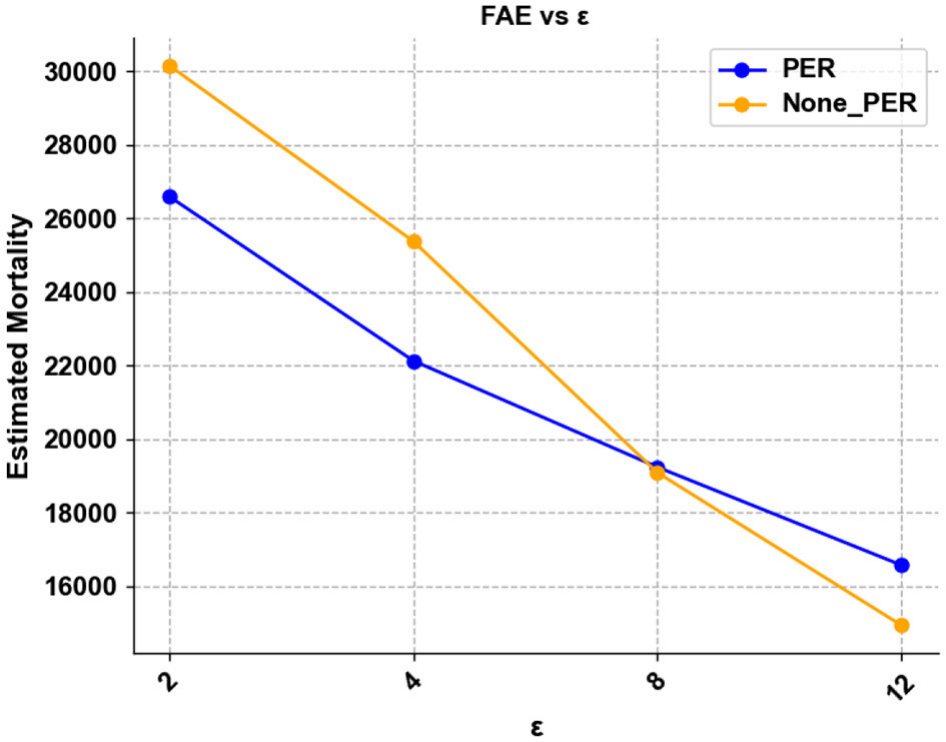
Showing the FAE for different ε.

Figure 5 demonstrates that as ϵ decreases, the learning time required to reach a specific survival rate becomes longer, implying that larger ϵ requires more training steps to obtain a better strategy. At the same time, we observe that PER still has the effect of accelerating convergence for larger ε, but instead has the opposite effect in the case of smaller ϵ. When ε = 12, the learning efficiency of using PER is higher, when ε = 8, there is almost no change, and when ε = 4 and ε = 2, adding PER will significantly reduce the learning efficiency. We believe that PER increases the effect of perturbations and thus causes the model to converge less easily at high privacy budgets. In subsequent experiments, we will not use preferential empirical replay at low ε.

### 5.3 Estimated mortality

The usability of the model can be visualized by the Estimated Mortality. A high usability should ensure that patients have a low mortality rate, and a lower mortality rate means that the model learns a better strategy. We first compare the performance of models satisfying (8, 10^−6^)−differential privacy in treating patients with different SOFA values.

**Figure 6:**
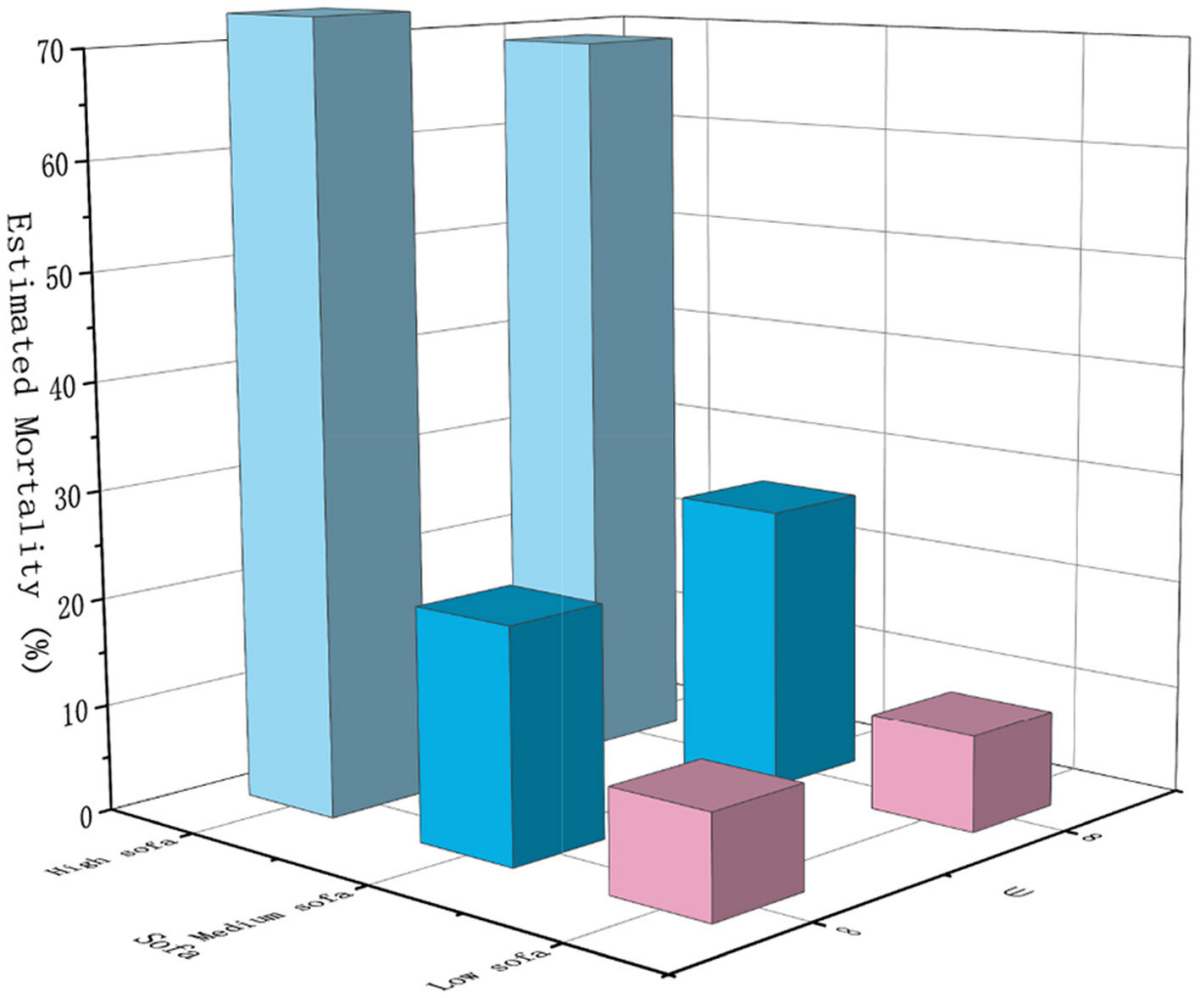
Estimated mortality rates of the model on different severity subcohorts at different privacy budgets (ε = ∞ indicates no differential privacy protection).

As shown in Figure 6, compared to the model without the inclusion of privacy protection, the model with the inclusion of privacy protection caused a change in the estimated mortality rate for both patients under the perturbation of the gradient, with a smaller effect on the treatment of patients with low SOFA, with only a 0.6% increase in the estimated mortality rate. In contrast, it had a larger impact on the treatment effect for patients with moderate SOFA and patients with high SOFA, with a 4.6% increase in estimated mortality for patients with moderate SOFA and, interestingly, an 4% decrease in estimated mortality for patients with a high SOFA instead, which may be due to the change in action selection. To make the data more representative, we observed the performance of the model when treating patients with moderate SOFA.

**Figure 7:**
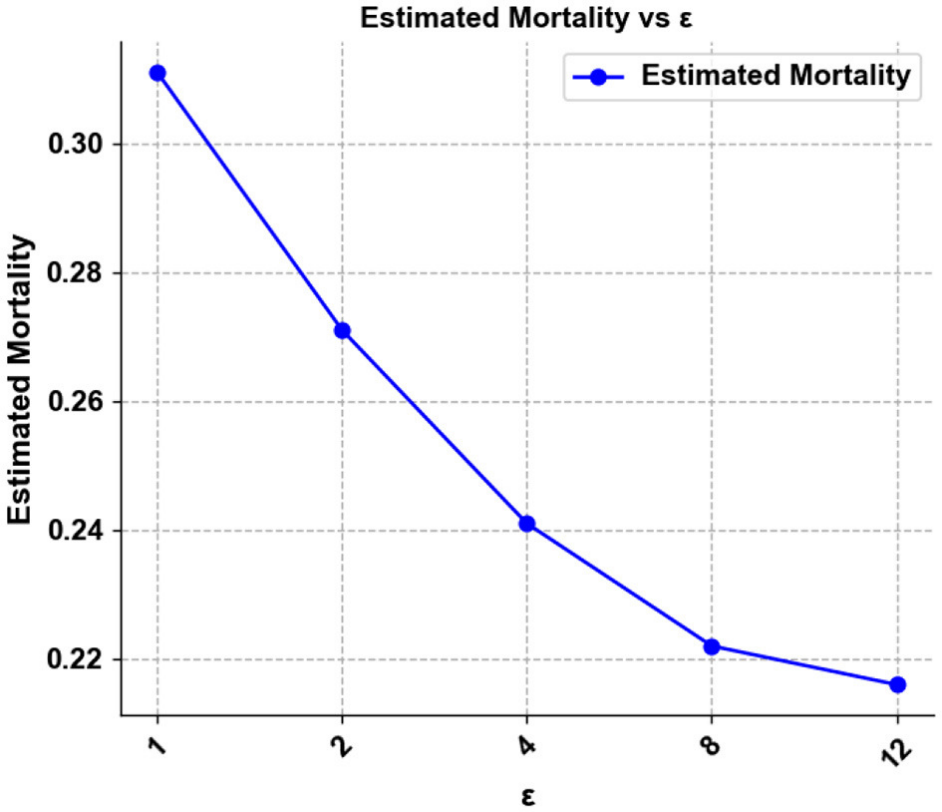
Model's estimated mortality for patients at different privacy budgets.

From Figure 7, it can be found that the mortality rate is negatively correlated with the privacy budget. The smaller the privacy budget, the higher the degree of privacy protection. As the privacy budget becomes smaller, the effect of the model becomes worse, and the mortality rate of patients increases. This means that a low privacy budget means poor usability. This is interpretable, to achieve tighter privacy protection, larger perturbations need to be added, which is bound to have an impact on model usability.

The slope of the polyline gets steeper and steeper with tighter privacy protections. The agent selects the action corresponding to the highest Q value, and the selection of the action affects the patient's mortality rate. Small parameter perturbation does not change the Q value greatly, so the action selection of the agent does not change greatly, and the impact on the mortality of patients is small. However, when the perturbation exceeds a certain threshold, the mortality rate of patients increases sharply.

Although using differential privacy protection will have an impact on usability, a balance between privacy and availability can be found by limiting the privacy budget. Our experiments also demonstrate that using Algorithm 1 can still provide strong privacy protection (ε < 10). Achieving high survival rates has a minimal impact on model performance.

## 6 Conclusion

In this paper, we investigated the use of differential privacy in reinforcement learning and accordingly proposed a privacy-preserving deep Q-network for secure sepsis treatment. The advantage of our approach is that the Gaussian noise is added to the gradient, thus achieving privacy preservation for reinforcement learning. Furthermore, we analyzed the impact of differential privacy on model learning efficiency and usability. Finally, we conducted experiments to evaluate our proposed method and the results confirmed that the proposed privacy-preserving deep Q-network could work for sepsis treatment without significant efficiency and accuracy drop. The experimental results also demonstrate that incorporating differential privacy into a deep reinforcement learning model helps clinicians to design personalized sepsis treatment strategies that effectively protect patient data privacy while simultaneously reducing sepsis-induced pulmonary tissue injury and respiratory dysfunction. In the future, we plan to explore the adaptive selection of related parameters. Under the premise of privacy, the model has higher usability.

## Data Availability

The original contributions presented in the study are included in the article/supplementary material, further inquiries can be directed to the corresponding author.
